# Metal-Chelating Macroalgal Extract as a Marine Antioxidant for Stabilizing DHA Nanoemulsions

**DOI:** 10.3390/antiox15010145

**Published:** 2026-01-22

**Authors:** Sakhi Ghelichi, Behdad Shokrollahi Yancheshmeh, Mona Hajfathalian, Seyed Hossein Helalat, Arpan Shrestha, Saroj Katwal, Charlotte Jacobsen

**Affiliations:** 1 Research Group for Bioactives–Analysis and Application, National Food Institute, Technical University of Denmark, 2800 Kongens Lyngby, Denmark; 2 Department of Health Technology, Technical University of Denmark, 2800 Kongens Lyngby, Denmark

**Keywords:** docosahexaenoic acid (DHA), nanoemulsion, lipid oxidation, metal chelation, *Palmaria palmata*, macroalgal extract

## Abstract

Docosahexaenoic acid (DHA), an omega-3 fatty acid essential for human health, is highly prone to oxidation in nanoemulsions due to their large interfacial area and presence of transition metal ions. This study investigated macroalgal chelators for stabilizing DHA-rich nanoemulsions. Sequential enzymatic–alkaline extraction using Alcalase^®^ produced an extract with the strongest Fe^2+^-chelating activity (IC_50_ = 1.22 mg/mL), protein content of 10.11 ± 0.15%, and total phenolics ≈ 17 µg GAE/mL. This extract was incorporated into nanoemulsions (5 wt% DHA oil, 1 wt% Tween^®^ 20) at 0.61, 1.22, and 2.44 mg/mL and compared with controls containing EDTA (0.025 mg/mL) or no antioxidant. Droplet size remained stable (D_3,2_ ≈ 77–80 nm; D_4,3_ ≈ 199–215 nm) and zeta potential averaged −17 to −19 mV, confirming physical stability. Confocal microscopy revealed concentration-dependent interfacial adsorption of extract components. During iron-accelerated storage, extract-treated nanoemulsions slowed hydroperoxide formation and delayed tocopherol depletion compared to the control, while reducing volatile oxidation markers such as 1-penten-3-ol by up to 40%. However, EDTA consistently provided superior protection against oxidation. These findings highlight the potential of macroalgal extracts as clean-label, natural chelators for mitigating metal-driven oxidation in DHA nanoemulsions, though synthetic chelators remain more effective under severe prooxidant conditions.

## 1. Introduction

Docosahexaenoic acid (DHA) is a nutritionally important omega-3 fatty acid that supports visual and neural function, helps regulate inflammatory and oxidative stress responses, and gives rise to bioactive lipid mediators essential for cellular health [1]. Because human metabolic pathways exhibit limited capacity to convert α-linolenic acid to DHA, adequate intake must be achieved primarily through direct consumption of preformed DHA [2]. As global interest in DHA-enriched functional foods continues to expand [3], ensuring the stability of this fatty acid during processing and storage has become an increasingly critical challenge. While marine fish oils have historically supplied much of the dietary DHA, their use is constrained by sustainability and contamination concerns [4]. Moreover, as plant-based diets become increasingly popular, fish-derived DHA sources are less suitable, making algal oil a sustainable and concentrated alternative that delivers DHA without relying on higher-trophic marine species [5].

Nevertheless, the high degree of unsaturation in DHA makes it highly susceptible to oxidative degradation, particularly in dispersed systems [6,7]. Oxidative degradation of DHA not only diminishes its nutritional value but also accelerates the formation of volatile oxidation products responsible for unpleasant sensory characteristics, thereby limiting its incorporation into emulsified food matrices. To overcome these barriers, nanoemulsion technology has been recommended as a delivery platform for DHA [8]. However, the high interfacial area characteristic of nanoemulsions can paradoxically intensify lipid oxidation, especially in the presence of transition metal ions [9]. This susceptibility highlights the importance of employing antioxidant strategies to effectively protect DHA in nanoemulsion systems.

In food systems, oxidative deterioration of unsaturated fatty acids is commonly mitigated using antioxidants that quench free radicals and agents that limit the catalytic activity of transition metals [10]. Metal chelators, often classified as secondary antioxidants, limit oxidative reactions by binding pro-oxidant transition metals and stabilizing their redox state, thereby restricting metal-mediated hydroperoxide breakdown and reducing interactions between metals and lipid substrates [11]. Their protective effects may arise through several mechanisms, including altering the redox behavior of metal ions and restricting their physical access to lipid substrates [12]. Synthetic chelating additives are commonly used in food formulations, with EDTA being particularly effective due to its strong metal-binding capacity and high efficacy at low concentrations and under acidic conditions [13]. However, increasing demand for clean-label ingredients has raised concerns regarding the use of synthetic chelators such as EDTA [14]. Moreover, the effectiveness of metal chelation is highly dependent on the characteristics of the food system itself. In emulsified systems, where the oil–water interface promotes close contact between pro-oxidant metals and lipid hydroperoxides, metal-catalyzed oxidation proceeds more rapidly than in low-moisture foods [13], indicating the need to carefully tailor chelating strategies to emulsion-based delivery systems such as DHA nanoemulsions.

Marine macroalgae have emerged as promising resources for functional food applications due to their rich and diverse composition of bioactive constituents [15]. These organisms provide a complex matrix of proteins, sulfated polysaccharides, and phenolic compounds that can contribute to both nutritional value and physicochemical functionality in food systems [16,17]. A growing body of research indicates that such components can interact at oil–water interfaces and enhance the oxidative stability of emulsified omega-3 lipids, including DHA, through multiple mechanisms such as antioxidant activity and interfacial stabilization [18,19]. Among red macroalgae, *Palmaria palmata* has attracted increasing interest as a sustainable source of food-grade bioactives, owing to its wide availability and established history of consumption [20]. Its use as a food ingredient is permitted under European Union regulations, supporting its suitability for incorporation into edible formulations [21]. Previous studies from our group have shown that extracts derived from this species using enzymatic [21], chemical [22], or combined extraction approaches [23] contain bioactive compounds with pronounced in vitro antioxidant capacities. However, antioxidant performance under simplified assay conditions does not necessarily translate to effectiveness in complex food matrices [24]. Consequently, evaluating the functionality of these seaweed extracts within real food-relevant systems, such as DHA-loaded nanoemulsions, is essential to determine their practical potential for oxidative stabilization.

Considering the susceptibility of DHA-rich nanoemulsions to metal-catalyzed oxidation and the growing interest in natural alternatives to synthetic chelators, this study investigated the metal-chelating and antioxidant potential of seaweed-derived extracts. Extracts were produced using a sequential enzymatic and alkaline extraction strategy with the use of different proteases and were initially screened for their Fe^2+^-chelating capacity in vitro. Comprehensive characterization was performed to relate chelating performance to compositional attributes, including protein content, degree of hydrolysis, amino acid composition, and total phenolic content. An extract exhibiting superior chelating activity was subsequently selected for incorporation into DHA-enriched nanoemulsions. Because the effectiveness of metal chelators is highly dependent on both the food matrix and the applied concentration [13], the selected extract was evaluated at three different concentrations and compared with a nanoemulsion without added antioxidant and a formulation containing EDTA as a synthetic reference. Interfacial tension measurements were conducted to assess the surface activity of the extracts at these concentrations, and confocal laser scanning microscopy (CLSM) was employed to visualize the microstructure of the resulting nanoemulsions. The physical stability of the nanoemulsions was assessed through measurements of droplet size, zeta potential, and viscosity, while oxidative stability was monitored during three weeks of storage under iron-accelerated conditions by tracking primary oxidation (peroxide value and tocopherol depletion) and secondary oxidation through measurement of formation of volatile oxidation products. This approach allows a comprehensive evaluation of the potential of seaweed extracts as natural, marine-derived metal chelators for stabilizing DHA nanoemulsions.

## 2. Materials and Methods

### 2.1. Macroalgal Biomass

*P. palmata* was harvested from the Faroe Islands during late autumn to early winter 2023 and obtained from a commercial supplier (Dansk Tang, Nykøbing Sjælland, Denmark). The biomass was freeze-dried (ScanVac CoolSafe, LaboGene A/S, Allerød, Denmark) to reduce moisture content, then milled to 0.5–1.0 cm particle size using a KN 295 Knifetec™ laboratory mill (Foss A/S, Hillerød, Denmark). The resulting powder was stored at −20 °C in sealed plastic bags protected from light until further use.

### 2.2. Chemicals, Enzymes, and Oil

Alcalase^®^ (2.4 amino acid units, AU-A/g), Flavourzyme^®^ (500–1000 leucine aminopeptidase units, LAPU/g), and Formea^®^ Prime (140 kilo mannitol units per gram, KMTU/g) were generously provided by Novenesis A/S (formerly Novozymes A/S, Bagsværd, Denmark). DHA-rich microalgal oil derived from *Schizochytrium* sp. was kindly supplied by Polaris (Quimper, France) and contained and 57.0 ± 0.3% DHA (C22:6). The oil also comprised α-, β-, γ-, and δ-tocopherols at 133 ± 0.9, 20 ± 0.3, 697 ± 2.9, and 302 ± 6.6 μg g^−1^, respectively. All solvents employed in analytical procedures were of HPLC grade (Lab-Scan, Dublin, Ireland), and amino acid standards were purchased from Sigma-Aldrich (St. Louis, MO, USA). Disodium ethylenediaminetetraacetate (EDTA) was obtained from Sigma-Aldrich (Steinheim, Germany). Additional reagents including ammonium bicarbonate, sodium acetate, imidazole, Tween^®^ 20, sodium hydroxide, sodium azide (NaN_3_), tocopherol standards (α, β, γ, δ), ammonium thiocyanate, barium dichloride (BaCl_2_·2H_2_O), iron chloride (FeCl_3_·6H_2_O), 96% ethanol, hydrogen peroxide, and volatile standards were also sourced from Sigma-Aldrich. All other chemicals were purchased from Merck (Darmstadt, Germany). High-purity water used in all experiments was produced at DTU Food using a Milli-Q^®^ Advantage A10 deionization system (Millipore Corporation, Billerica, MA, USA).

### 2.3. Extraction Method for Macroalgal Biomass

Extracts were obtained from *P. palmata* biomass using a sequential enzymatic and alkaline extraction procedure, employing different combinations of commercial proteolytic enzymes. In brief, 4 g of freeze-dried biomass was rehydrated in distilled water at a 1:20 *w*/*v* ratio and incubated at 50 °C for 1 h. Since the initial pH of the biomass–water mixture was suitable for enzymatic activity (approximately 6–8), no pH adjustment was needed. Enzymatic extraction was carried out with three proteases including Alcalase^®^, Flavourzyme^®^, or Formea^®^ Prime applied either individually at 2% of the protein content (dry weight basis) or in binary combinations, with each enzyme added at 1% of the protein content (dry weight basis). The extractions were conducted in a shaking water bath (SW 22, Julabo GmbH, Seelbach, Germany) at 50 °C and 80 rpm for 14 h. Enzyme activity was terminated by heating the mixtures to 95 °C for 20 min. The resulting suspensions were filtered through a 1 mm mesh filter, and the liquid enzymatic extracts were collected in blue-capped bottles and stored at 4 °C. The remaining solids from each enzymatic treatment were subjected to a three-step alkaline extraction using 80 mL of a solution containing 1 g/L N-acetyl-L-cysteine (NAC) and 4 g/L sodium hydroxide (NaOH). Each extraction was performed on an orbital shaker at 130 rpm at room temperature for 1.5 h, with fresh alkaline solution used for each step. Solid residues were transferred between rounds, and all three alkaline extracts were pooled with the corresponding enzymatic extract. For simplicity, the combined enzymatic/alkaline extracts were coded based on the enzyme(s) used: Ac (Alcalase^®^), Fz (Flavourzyme^®^), Fp (Formea^®^ Prime), Ac-Fz (Alcalase^®^ + Flavourzyme^®^), Ac-Fp (Alcalase^®^ + Formea^®^), Fz-Fp (Flavourzyme^®^ + Formea^®^), and NoE (no enzyme). The pH of each pooled extract was adjusted to 8.5–9.0 to ensure complete solubilization of proteins, peptides, and amino acids. All extracts and residual solids were pre-frozen at −20 °C for 2 h, followed by −80 °C for 24 h, before freeze-drying (LaboGene A/S, Allerød, Denmark). The resulting powders were stored in sealed zip-lock bags at −80 °C until further analysis. All extractions were performed in duplicate. Mass balances were calculated by accurately weighing all samples using an analytical balance with 0.01 g readability.

### 2.4. Characterization of Extracts

#### 2.4.1. Protein Content and Recovery

The total protein in the enzymatic and alkaline extracts was quantified by determining total nitrogen using the Dumas combustion method with a Vario EL Cube analyzer (Elementar Analysensysteme GmbH, Langenselbold, Germany). Protein concentrations were calculated by multiplying the measured nitrogen content by a conversion factor of 5.0 [15]. Protein recovery in the extracts was determined using the following equation:

(1) $$Protein\;recovery\left(\%\right)=\frac{M_{E}\times P_{E}}{M_{B}\times P_{B}}\times 100$$

where $M_{E}$ and $P_{E}$ denote the mass and protein content of the extract, and $M_{B}$ and $P_{B}$ correspond to the mass and protein content of the biomass, respectively.

#### 2.4.2. Degree of Hydrolysis (DH)

The extent of protein hydrolysis in the extracts was evaluated using the o-phthaldialdehyde (OPA) assay. The OPA reagent was prepared according to the protocol described in [21]. Extracts were diluted to achieve protein concentrations ranging from 0.05% to 0.25% and subsequently mixed with the OPA reagent in a microplate format. Absorbance was measured at 340 nm, and sample concentrations were determined using a calibration curve generated with L-serine. Serine equivalents were calculated as follows:

(2) $$Serine\;equivalents\left(mg\;Ser/mL\right)=\;\frac{\left(A_{E}-\;A_{B}\right)-intercept}{Slope}\;\times dilution\;factor$$

where $A_{E}$ and $A_{B}$ denote the absorbance of the extract and blank, respectively, and the slope and intercept were obtained from the L-serine standard curve. The degree of hydrolysis was then calculated using

(3) $$DH\;\left(\%\right)=\frac{E}{P\times 10}\times 100$$

where E represents the serine equivalents of the extract (mg Ser/mL) and P is the protein content expressed as a percentage. All measurements were performed in duplicate.

#### 2.4.3. Total Phenolic Content (TPC)

The total phenolic content of the extracts was measured using the Folin–Ciocalteu method [25]. In this assay, extracts were mixed with the Folin–Ciocalteu reagent and allowed to react at room temperature for 5 min, followed by the addition of 6% sodium bicarbonate. The reaction mixtures were incubated for 60 min at ambient temperature, after which absorbance was recorded at 725 nm using a UV-visible spectrophotometer (Shimadzu UV mini 1240, Duisburg, Germany). TPC was expressed as micrograms of gallic acid equivalents (µg GAE) per mL of extract and calculated using the following equation:

(4) $$TPC\;\left(µg\;GAE/mL\;extract\right)=\;\frac{\left(A-I\right)}{S}$$

where A is the measured absorbance of the sample, and S and I are the slope (0.007) and intercept (0.021) of the gallic acid calibration curve, respectively.

#### 2.4.4. Amino Acid Profile

Approximately 30 mg of each sample was subjected to hydrolysis using 6 M HCl at 110 °C for 18 h. The hydrolyzed samples were filtered through 0.22 µm cellulose acetate syringe filters into 4 mL vials, and 100 µL portions were transferred to fresh vials. The pH of the aliquots was adjusted by sequentially adding 1.5 mL of 0.2 M KOH and 1.6 mL of 100 mM ammonium acetate buffer (pH 3.1, adjusted with formic acid), achieving a final dilution factor of 32. Amino acid profiles were analyzed using liquid chromatography–mass spectrometry (LC-MS) on an Agilent 1260 Infinity II Series system coupled to a Quadrupole 6120 MS with an electrospray ionization (ESI) source (Agilent Technologies, Santa Clara, CA, USA). Mass spectrometric detection was carried out in positive ion mode, with a drying gas temperature of 300 °C, drying gas flow of 7.0 L/min, and a nebulizer pressure of 15 psi. Separation was carried out on a BioZen 2.6 µm Glycan column (100 × 2.1 mm, Phenomenex, Torrance, CA, USA) at 40 °C, with a flow rate of 0.5 mL/min, injection volume of 1 µL, and a total run time of 16 min. A gradient elution was employed using mobile phase A (10 mM ammonium formate in acetonitrile) and mobile phase B (10 mM ammonium formate in Milli-Q water) as follows: 0–2 min, 0–5% B; 2–7 min, 5–20% B; 7–8 min, 20–80% B; 12.1 min, 0% B; 12.1–16 min, 0% B. Quantification was performed using MassHunter Quantitative Analysis v7.0 (Agilent Technologies) with calibration curves prepared from a mixture of 17 amino acids at five different concentrations. It should be noted that during hydrolysis, glutamine and asparagine were converted into glutamic acid and aspartic acid, respectively, whereas tryptophan and cysteine were degraded and therefore could not be detected.

#### 2.4.5. Fe^2+^ Chelating Ability

The ability of the extracts to chelate Fe^2+^ ions was assessed using a modified version of the method described by [26], adapted for microplate measurements. In brief, 100 µL of each extract solution was combined with 110 µL of distilled water and 20 µL of 2 mM FeCl_2_. After 3 min, 20 µL of 5 mM ferrozine was added to initiate complex formation, and the mixture was thoroughly homogenized. The samples were then incubated at room temperature for 10 min, followed by absorbance measurement at 562 nm. Distilled water served as the blank, while control samples were prepared without Fe^2+^ and ferrozine. All measurements were performed in triplicate, and 0.06 mM EDTA was used as a positive control. The percentage of Fe^2+^ chelation was calculated using the following equation:

(5) $${Fe}^{2+}\;chelating\;(\%)=\left(1-\frac{\left(A_{E}-A_{C}\right)}{A_{B}}\right)\times 100$$

where $A_{E}$, $A_{C}$ and $A_{B}$ correspond to the absorbances of the extract, control, and blank, respectively. The IC_50_ values were determined from linear regression of the dose–response curves.

#### 2.4.6. Dynamic Interfacial Tension (IFT)

The interfacial properties of the extract with the highest metal chelating activity were evaluated by monitoring IFT at the oil–water interface. Solutions of this extract were prepared in 10 mM sodium acetate-imidazole buffer (pH 7) at three different concentrations corresponding to those used in the emulsion study. Pendant drops were formed on MCT oil and imaged every 2 s over a 30 min period using an automated drop tensiometer (OCA25, DataPhysics Instruments GmbH, Filderstadt, Germany) at 25 °C. IFT values were calculated using the Young-Laplace equation, and the temporal changes were analyzed to assess adsorption kinetics. For comparison, reference measurements were performed with buffer alone and Milli-Q water.

### 2.5. Preparation of Nanoemulsions

Oil-in-water nanoemulsions were prepared to evaluate the metal-chelating effects of the seaweed extracts. Only the extract exhibiting the highest Fe^2+^-chelating activity was used, tested at three concentrations based on its IC_50_: 0.61 mg/mL (half IC_50_), 1.22 mg/mL (IC_50_), and 2.44 mg/mL (twice IC_50_). Aqueous solutions of the extract were prepared in 10 mM sodium acetate-10 mM imidazole buffer (pH 7). EDTA (0.025 mg/mL) served as positive control, and a solution without any antioxidant was used as a negative control. Nanoemulsions (220 g) were formulated with 5 wt% DHA-rich algal oil and 1 wt% Tween^®^ 20. Pre-emulsification was carried out using a high-shear mixer (Ultraturrax, Ystral, Ballrechten-Dottingen, Germany) at 16,000 rpm for 3 min, during which the oil phase was gradually added. Final homogenization was performed with a microfluidizer (M110L, Microfluidics, Westwood, MA, USA) equipped with a ceramic interaction chamber (CIXC, F20Y, 75 µm) at 9000 psi for three passes. Sodium azide (0.05 wt%) and 50 μM FeSO_4_ were added as preservatives and iron-induced oxidation accelerator, respectively. The nanoemulsions were labeled as follows: E1 (negative control, no antioxidant), E2 (EDTA, positive control), E3 (extract at 0.61 mg/mL, 0.5 × IC_50_), E4 (extract at 1.22 mg/mL, IC_50_), and E5 (extract at 2.44 mg/mL, 2 × IC_50_). Nanoemulsions were stored at room temperature (19–20 °C) in the dark for up to 8 days. Samples for lipid oxidation analysis were collected on days 0, 2, 4, 6, and 8, transferred into amber bottles, flushed with nitrogen, and stored at −40 °C until further use.

### 2.6. Characterization of Nanoemulsions

#### 2.6.1. Droplet Size Distribution

The droplet size distribution of the nanoemulsions was assessed on days 1 and 8 using a laser diffraction system (Mastersizer 2000, Malvern Instruments Ltd., Malvern, Worcestershire, UK). Prior to analysis, samples were diluted with circulating water at 3000 rpm until an obscuration of 12–15% was achieved. Refractive indices of 1.469 and 1.330 were applied for the oil and aqueous phases, respectively. Particle size was reported as the surface-area mean diameter (D_3__,2_) and volume mean diameter (D_4,3_). All measurements were performed in duplicate.

#### 2.6.2. Zeta Potential

On the second day, the zeta potential of the nanoemulsion droplets was determined using a Zetasizer Nano ZS (Malvern Instruments Ltd., Worcestershire, UK). For each measurement, 0.032 g of the emulsion was precisely weighed and diluted with 40 g of distilled water, followed by thorough mixing with a vortex mixer. The prepared samples were then placed into disposable folded capillary cells (DTS-1070, Malvern Instruments Ltd., UK). Each analysis was carried out in duplicate at 25 °C, covering a zeta potential range from −100 to +50 mV, with 100 runs per measurement. Based on previous studies in our laboratory, which showed no significant changes in zeta potential over the short storage period, we focused on early measurements (day 2) and did not extend zeta potential analysis to day 8.

#### 2.6.3. Apparent Viscosity

The apparent viscosity of the samples was assessed using a stress-controlled rheometer (Stresstech, Reologica Instruments AB, Lund, Sweden) equipped with a CC25 bob-and-cup configuration. Measurements were conducted at 25 °C over a shear stress range of 0.0025–2 Pa. Viscosity values were recorded at a shear rate of 100 s^−1^ and reported in centipoise (cP).

#### 2.6.4. CLSM

CLSM was utilized to examine the microstructure of nanoemulsions. Samples were handled carefully to preserve droplet morphology and prevent aggregation or disruption. Oil and protein droplets were stained simultaneously using Nile Red and Nile Blue, respectively. Both dyes were added to the samples at the same time and incubated for 5 min at room temperature prior to imaging to allow adequate staining. Nile Red was used for lipid staining and selectively labeled neutral lipid-rich oil droplets, while Nile Blue was used to label protein-containing structures. No washing steps were performed after dye addition to avoid perturbation of droplet structure and spatial organization. Fluorescence imaging was performed using a Zeiss LSM 900 Airyscan confocal laser scanning microscope (Oberkochen, Germany), operated with ZEN Blue Edition software (version 2.6). Images were acquired using a 60× oil-immersion objective. Nile Red fluorescence was excited using a 488 nm laser line, and emission was collected at 509 nm, producing green fluorescence corresponding to lipid droplets. Nile Blue fluorescence was excited at 627 nm, and emission was collected at 623 nm, producing red fluorescence associated with protein-rich droplets. For all experiments, the detector gain was set to 650 V and kept constant across all samples. Post-acquisition processing was limited to linear adjustments of brightness and contrast applied uniformly across all images. No non-linear processing or artificial signal enhancement was performed.

#### 2.6.5. Peroxide Value (PV)

Lipids were extracted from the emulsions in duplicate using a modified Bligh and Dyer method [27], which minimized the amount of chloroform/methanol (1:1, *w*/*w*) required. The peroxide value of the lipid extracts was determined in duplicate using the ferrothiocyanate colorimetric assay, with absorbance measured at 500 nm. While the original method described by Shantha and Decker [28] uses cumene hydroperoxide for calibration and expresses PV as mmol peroxide/kg lipid, FeCl_3_ was used in the present study to generate the standard curve, and PV was expressed as meq peroxide/kg lipid.

#### 2.6.6. Tocopherol Depletion

Tocopherol concentrations in the lipid extracts were analyzed using normal-phase high-performance liquid chromatography (HPLC) following AOCS Official Method Ce 8–89 [29]. The analyses were performed on an Agilent 1100 series HPLC system under isocratic conditions with a mobile phase of heptane and 2-propanol (100:0.4, *v*/*v*) at a flow rate of 1.0 mL/min and an injection volume of 20 µL. Separation was achieved using a Waters Spherisorb silica column (3 µm, 4.6 mm × 150 mm) coupled with a guard column (5 µm, 4.6 mm × 10 mm). Fluorescence detection was set at 290 nm for excitation and 330 nm for emission. Approximately 2 g of each extract was dissolved in 1 mL of heptane prior to injection. Tocopherols were quantified using external standards with single-point calibration. All measurements were performed in duplicate, and results were expressed as micrograms of individual tocopherols per gram of oil.

#### 2.6.7. Volatile Secondary Oxidation Compounds

Volatile secondary oxidation compounds were quantified using an automated dynamic headspace sampler (Gerstel GmbH & Co. KG, Mülheim an der Ruhr, Germany) coupled with gas chromatography-mass spectrometry (GC–MS) (Agilent 6890 N/5973, USA) according to the method described by Thomsen et al. [30] Approximately 1 g of sample was added with 4-methyl-1-pentanol (30 µg/g) as an internal standard, mixed thoroughly, and incubated at 60 °C for 4 min with intermittent agitation. Headspace volatiles were purged with nitrogen (50 mL/min for 20 min), captured on Tenax GR 300 sorbent tubes, dried, and thermally desorbed into the GC system. Separation was achieved on a DB-1701 capillary column (30 m × 0.25 mm ID, 0.5 µm film thickness) using a temperature program from 35 °C to 240 °C in stepwise increments. Mass spectrometric detection was performed in electron ionization mode (70 eV) scanning m/z 30–250. Compound identification relied on the Wiley 138K spectral library, and quantification was based on 6-point calibration curves (1–250 μg/mL) prepared from authentic external standards under identical conditions. The compounds quantified included 2-ethylfuran, 4-methyl-1-pentanol, 1-penten-3-one, 1-penten-3-ol, hexanal, and (*E*,*E*)-2,4-heptadienal. Each emulsion was analyzed in triplicate.

### 2.7. Statistical Analysis

Statistical analyses were performed using one-way Analysis of Variance (ANOVA), and differences among means were evaluated with Bonferroni’s post hoc test. Paired *t*-tests were applied to compare droplet size between Day 1 and Day 8 for each emulsion. All computations were carried out in OriginPro 2023 (OriginLab Corporation, Northampton, MA, USA). A significance level of *p* < 0.05 was adopted for all tests.

## 3. Results and Discussion

### 3.1. Characteristics of Macroalgal Extracts

#### 3.1.1. Protein Content and Recovery and DH

Table 1 presents the protein content and recovery of extracts and solid residues, as well as the DH for all seven treatments. Alcalase^®^ (Ac) achieved the highest protein content in extracts (10.11 ± 0.15%), significantly surpassing all other treatments (*p* < 0.05), confirming its superior ability to solubilize seaweed proteins into the water-soluble fraction [21]. Consistently, the protein content of solid residues after Alcalase^®^ treatment was among the lowest (14.17 ± 0.79%), indicating more extensive protein removal from the seaweed matrix. Protein recovery in extracts followed a similar trend: Alcalase^®^ alone yielded ~95%, significantly higher than all other treatments (*p* < 0.05). Dual-enzyme combinations (Ac-Fz and Ac-Fp) resulted in recovery of approximately 81–82%, which was lower than that obtained with Alcalase^®^ alone (94.99%), but still higher than single Flavourzyme^®^ or Formea^®^ Prime treatments (circa 61–70%). The lower recovery in the dual-enzyme treatments is likely due to the reduced concentration of each enzyme (1% each) compared to 2% when Alcalase^®^ was used alone, suggesting that 2% Alcalase^®^ is required to achieve optimal protein recovery. In contrast, NoE (no enzyme) resulted in the lowest recovery (53.65%), highlighting the critical role of enzymatic hydrolysis in protein extraction.

The highest DH was observed for Ac-Fz (45.76 ± 8.36%), followed by Fz-Fp (34.50 ± 4.07%) and Alcalase^®^ alone (30.90 ± 3.22%). These results indicate that combining Alcalase^®^ and Flavourzyme^®^ significantly enhances hydrolysis compared to single-enzyme treatments (*p* < 0.05). However, protein recovery with this combination did not exceed that obtained with Alcalase^®^ alone. This is likely because, although Flavourzyme^®^ efficiently cleaves peptides into shorter chains and increases DH, shorter peptides and free amino acids may be lost during extraction, limiting total protein recovery. Similar trends were observed in our previous studies, where extensive hydrolysis was achieved with Flavourzyme^®^-containing treatments despite lower protein content in the extracts [21]. Formea^®^ Prime alone exhibited the lowest DH (22.91 ± 4.31%), consistent with its classification as a mild protease (manufacturer’s data). The strong hydrolytic performance of Alcalase^®^ and Flavourzyme^®^ can be attributed to their broad substrate specificity and multiple catalytic sites, which facilitate extensive cleavage of peptide bonds [31,32]. Overall, Alcalase^®^ demonstrated superior protein solubilization and recovery, while dual-enzyme systems, particularly Ac-Fz, achieved the highest degree of hydrolysis.

#### 3.1.2. TPC

**Figure 1:**
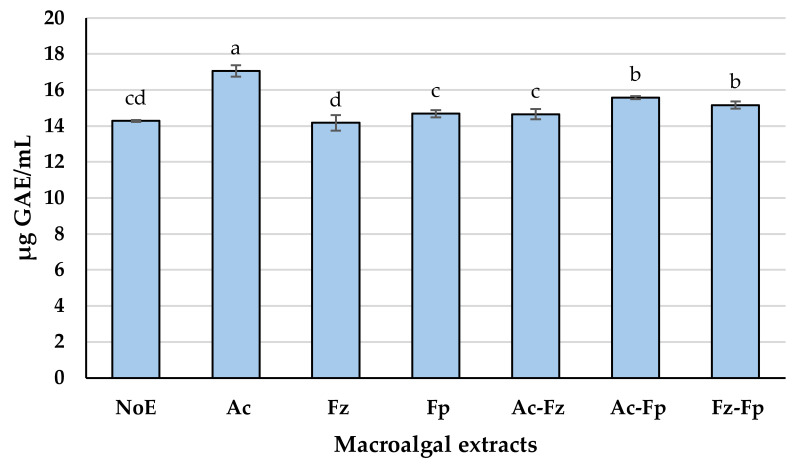
Total phenolic compounds (TPC) of macroalgal extracts obtained through sequential enzymatic and alkaline procedure. Data were expressed as mean ± standard deviation (n = 2, representing independent experimental replicates). Different letters indicate significant differences among treatments (*p* < 0.05). NoE (no enzyme), Ac (Alcalase^®^), Fz (Flavourzyme^®^), Fp (Formea^®^ Prime), Ac-Fz (Alcalase^®^ + Flavourzyme^®^), Ac-Fp (Alcalase^®^ + Formea^®^ Prime), and Fz-Fp (Flavourzyme^®^ + Formea^®^ Prime), each followed by alkaline extraction.

Significant differences were observed among TPC of the macroalgal extracts (*p* < 0.05) (Figure 1). Ac exhibited the highest TPC (≈17 µg GAE/mL), clearly outperforming all other treatments. This indicates that Alcalase^®^ is particularly effective in promoting the release of phenolic compounds from the seaweed matrix when combined with alkaline extraction. Two combined-enzyme treatments, Ac-Fp and Fz-Fp, resulted in intermediate TPC values (≈15–15.6 µg GAE/mL), significantly lower than Ac but higher than the remaining treatments (*p* < 0.05). These results suggest that adding Formea^®^ Prime to Alcalase^®^ or Flavourzyme^®^ provides some benefit, although not as pronounced as using Alcalase^®^ alone. Fp and Ac-Fz yielded TPC values around 14.7 µg GAE/mL, which were significantly lower than Ac and the two combination treatments mentioned above (*p* < 0.05). The control (NoE) and Fz showed the lowest TPC (≈14.2 µg GAE/mL), indicating minimal improvement compared to untreated samples. The superior performance of Alcalase^®^ may be attributed to its higher cell-wall-disrupting activity, which promotes the release of phenolic compounds bound to structural polysaccharides. This observation is consistent with previous reports highlighting the strong role of Alcalase^®^ in enhancing TPC in macroalgal extracts [33]. The subsequent alkaline extraction likely enhances solubilization of these compounds into the aqueous phase, as previously reported [34]. Since most macroalgal polyphenols are associated with cell wall components, their release largely depends on the extent of cell wall degradation [35], which appears to be more effectively achieved when Alcalase^®^ is used as the enzymatic treatment.

#### 3.1.3. Amino Acid Composition

Table 2 summarizes the amino acid composition of the macroalgal extracts. Except for phenylalanine, histidine, and cystine, most amino acids were significantly higher in Ac compared to all other treatments (*p* < 0.05), confirming the strong hydrolytic efficiency of Alcalase^®^ in releasing amino acids into the water-soluble fraction. This trend was particularly evident for leucine, isoleucine, valine, and alanine, which are key contributors to nutritional quality and flavor development. The total amino acid (TAA) content was highest in Fz-Fp (111.77 mg/g), followed closely by Ac-Fz and Ac (102.33 and 100.19 mg/g, respectively), indicating that combining proteases can enhance overall protein solubilization. However, Alcalase^®^ alone still provided a competitive yield compared to dual-enzyme systems. Notably, the order of TAA content does not fully match the protein contents measured by the Dumas method, which showed higher values for Alcalase^®^ extracts. This discrepancy could be due to several factors, for instance loss of tryptophan or other labile amino acids during hydrolysis, or the release of non-protein nitrogen-containing compounds that are detected by the Dumas method but not counted in TAA analysis. Essential amino acids (EAA) followed a similar pattern, with Ac showing the highest EAA/TAA ratio (0.33), suggesting a superior nutritional profile compared to other treatments. This aligns with previous findings that Alcalase^®^ preferentially liberates essential residues [21].

Methionine levels were significantly higher in Ac (2.06 mg/g) than in other treatments (*p* < 0.05), which is nutritionally relevant since methionine and histidine are limiting essential amino acids in macroalgal proteins [15]. The elevated methionine content in Alcalase^®^-treated extracts reinforces its potential for improving amino acid balance in seaweed-derived ingredients. A striking observation was the cystine distribution. While Alcalase^®^-treated extracts, either alone or in combination with other two proteases, contained maximum ~16 mg/g cystine, treatments involving Flavourzyme^®^ or Formea^®^ Prime exhibited extremely high cystine levels (68–84 mg/g). This discrepancy is unexpected given the uniform biomass source. One plausible explanation is that Alcalase^®^’s broad specificity and aggressive cleavage pattern released more overall protein but retained disulfide-rich regions in insoluble fragments, whereas Flavourzyme^®^ and Formea^®^ Prime generated smaller peptides enriched in cystine that remained soluble. The exact mechanism behind this phenomenon warrants further investigation, as it may involve enzyme-specific cleavage preferences and peptide solubility dynamics. Overall, Alcalase^®^ effectively enhanced EAA availability, while dual-enzyme treatments maximized TAA recovery in the extracts, highlighting the role of enzyme selection in optimizing macroalgal extract functionality.

#### 3.1.4. Metal Chelating Ability

**Figure 2:**
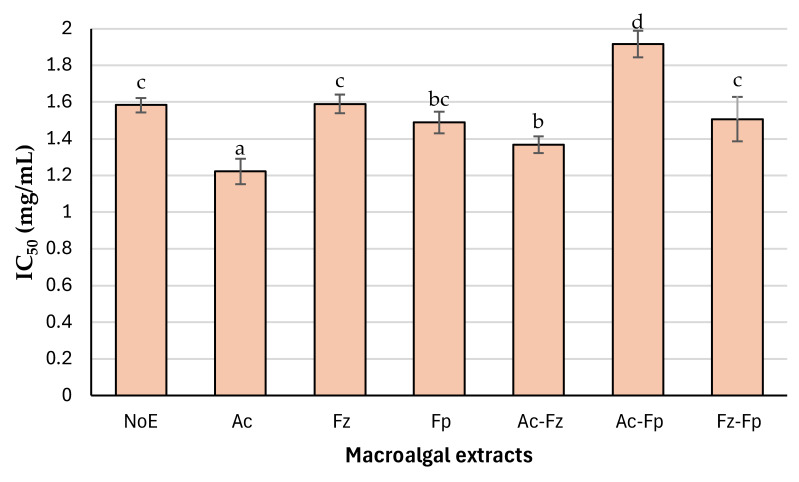
In vitro metal ion chelating activities of macroalgal extracts obtained through sequential enzymatic and alkaline procedure. Data were expressed as mean ± standard deviation (n = 6, representing measurements from two independent extracts, each analyzed in triplicate). Different letters indicate significant differences among treatments (*p* < 0.05). NoE (no enzyme), Ac (Alcalase^®^), Fz (Flavourzyme^®^), Fp (Formea^®^ Prime), Ac-Fz (Alcalase^®^ + Flavourzyme^®^), Ac-Fp (Alcalase^®^ + Formea^®^ Prime), and Fz-Fp (Flavourzyme^®^ + Formea^®^ Prime), each followed by alkaline extraction.

The Fe^2+^ chelating capacity of the macroalgal extracts, expressed as IC_50_ values, revealed clear differences among treatments (Figure 2). Ac demonstrated the strongest chelating ability, with an IC_50_ of 1.22 mg/mL, significantly lower than all other treatments (*p* < 0.05). This indicates the presence of components in Ac effective in binding ferrous ions. At the opposite end, Ac-Fp exhibited the weakest chelating activity (IC_50_ ≈ 1.92 mg/mL), suggesting that adding Formea^®^ Prime to Alcalase^®^ may not enhance, and could even hinder, the formation of chelating-active molecules under the tested conditions. Intermediate IC_50_ values were observed for Fz, NoE, and Fz-Fp (≈1.50–1.59 mg/mL), while Fp and Ac-Fz achieved slightly better performance (≈1.36–1.49 mg/mL), though still significantly less effective than Ac (*p* < 0.05). This may be linked to ability of Alcalase^®^ to generate peptides enriched in acidic amino acids such as glutamic acid (Table 2), which are known to form stable complexes with Fe^2+^ ions [36]. Additionally, Alcalase^®^ treatment likely promotes the release of phenolic compounds (Figure 1), which can contribute to metal ion chelation when combined with alkaline extraction [37]. The synergy between peptides and phenolics may explain the enhanced activity observed for Ac, whereas treatments lacking this combination showed weaker performance. In addition, the formation of binary or ternary covalent conjugates or noncovalent complexes of peptides, polyphenols, and polysaccharides during Alcalase^®^ treatment may enhance the antioxidant properties of Ac, because such interactions have been reported to improve antioxidant activity [38]. Considering the significantly better metal chelating properties of Ac (*p* < 0.05), this extract was selected for testing in DHA oil-in-water nanoemulsion.

#### 3.1.5. IFT

**Figure 3:**
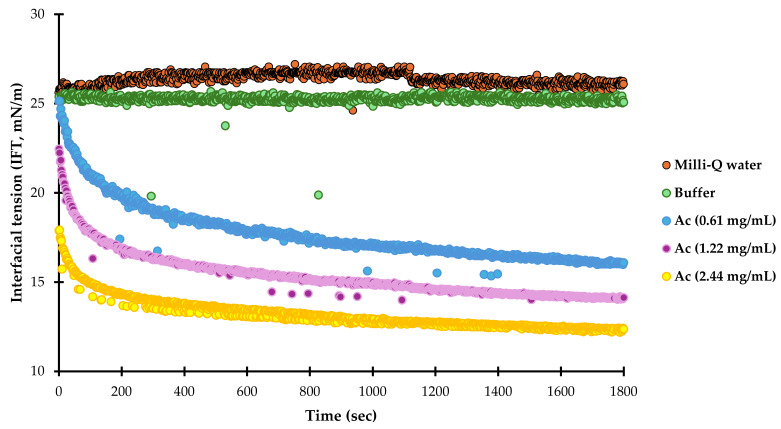
Dynamic interfacial tension (mN/m) between MCT oil and Milli-Q water, sodium acetate-imidazole buffer, and Alcalase^®^/alkaline macroalgal extract at three different concentrations.

The dynamic interfacial tension at the oil–water interface was monitored for Milli-Q water, buffer solution, and Alcalase^®^ extract (Ac) at three concentrations corresponding to those tested in nanoemulsion formulations (Section 2.5) (Figure 3). As expected, the IFT of Milli-Q water and buffer remained nearly constant over time (≈26 and 25 mN/m, respectively), confirming the absence of surface-active components in these systems. In contrast, the presence of Ac caused a pronounced reduction in IFT, and the extent of this reduction was strongly concentration dependent. At the lowest concentration (0.61 mg/mL), the IFT decreased from ~25 mN/m to ~19 mN/m within the first few minutes, whereas at 1.22 mg/mL and 2.44 mg/mL, the final IFT values reached ~14 and ~12 mN/m, respectively. This rapid decline followed by stabilization indicates favorable adsorption kinetics, suggesting that peptides and other surface-active molecules in the extract quickly migrate and rearrange at the oil–water interface [39]. This dose-dependent effect highlights the role of molecular composition and availability of amphiphilic peptides in interfacial activity. Alcalase^®^ hydrolysis likely generates peptides with hydrophobic and hydrophilic domains, enabling them to act as natural emulsifiers. Additionally, co-extracted polysaccharides and phenolic compounds may contribute to interfacial behavior, as previously reported for seaweed-derived extracts [40]. The observed reduction in IFT is likely mediated by multiple mechanisms: amphiphilic peptides adsorb at the interface through their hydrophobic and hydrophilic domains, while polyphenols and polysaccharides may interact with these peptides via hydrogen bonding or hydrophobic interactions, enhancing the cohesion and stability of the interfacial layer [38]. These interactions help the extract components form a dynamic, partially packed interfacial layer that can contribute to emulsion stabilization when combined with surfactants in later formulations. The ability of Ac extract to significantly lower IFT suggests its potential to improve emulsion stability; however, the final IFT values remain higher than those typically achieved with synthetic surfactants (lower than 5 mN/m) [41], indicating that additional stabilizing strategies may still be required for commercial applications.

### 3.2. Physical Stability of Nanoemulsions

Zeta potential values of the nanoemulsions are reported in Table 3. On Day 1, zeta potential ranged from −17.25 ± 0.49 mV in E4 to −18.95 ± 0.21 mV in E2, with no significant differences among the samples (*p* > 0.05). The relatively small variation in zeta potential suggests that the addition of Ac at different concentrations did not appreciably alter the surface charge of the droplets. This is likely because the extract components either did not adsorb to the oil–water interface or carried insufficient charge to influence droplet potential. The main contributor to the overall surface charge of the emulsifier-coated oil droplets could be Tween^®^ 20. Although Tween^®^ 20 is a nonionic surfactant, previous studies have shown that oil droplets coated by it can exhibit a measurable negative charge, attributed to the presence of surface-active anionic impurities in the surfactant and/or oil phase (such as free fatty acids or phospholipids) [42]. Even small amounts of these substances can impart an appreciable surface charge. Overall, the moderate negative charge on the droplets should contribute to good colloidal stability by generating electrostatic repulsion between oil droplets [43], complemented by steric repulsion from the hydrophilic head groups of Tween^®^ 20 [44].

D_3,2_ and D_4,3_ values of the oil droplets were measured at Day 1 and Day 8 (Table 3). At Day 1, D_3,2_ values ranged from 77.65 nm in E5 to 80.25 nm in E3, with significant differences among samples (*p* < 0.05). Similarly, D_4,3_ values ranged from 199.0 nm in E4 to 215.0 nm in E2 and E3. After 8 days of storage, droplet sizes remained relatively stable, with only minor changes observed. These results indicate that the nanoemulsions exhibited good physical stability during storage. The slight differences in droplet size among samples are unlikely to be due to interfacial competition, as the Ac extract components were probably not sufficiently amphiphilic to displace Tween^®^ 20 from the oil–water interface. Instead, the variations may reflect minor differences in formulation or measurement variability. Similar trends were observed for D_4,3_ values, which also showed minimal changes over time. These findings are consistent with the excellent emulsifying properties of Tween^®^ 20, which rapidly adsorbs to oil–water interfaces during homogenization and forms a protective coating around droplets [34], which inhibits flocculation and coalescence by generating strong steric and electrostatic repulsive forces.

All formulations exhibited non-Newtonian shear-thinning behavior typical of dilute emulsions, with apparent viscosity decreasing as shear rate increased. The apparent viscosity of the nanoemulsions at 100 s^−1^ ranged from 1.34 to 1.47 cP on Day 1 and 1.16 to 1.41 cP on Day 8, with no significant differences among samples (*p* > 0.05). All emulsions exhibited low viscosity typical of dilute systems, and the slight decrease observed during storage is unlikely to be caused by droplet aggregation, given the stable size profiles. Overall, the combination of moderate negative zeta potential, small and stable droplet sizes, and low viscosity indicates that the DHA nanoemulsions were physically stable throughout storage. Incorporation of Ac extract at different concentrations did not compromise these properties, supporting its suitability for subsequent oxidative stability evaluations.

### 3.3. Microstructure of Nanoemulsions

**Figure 4:**
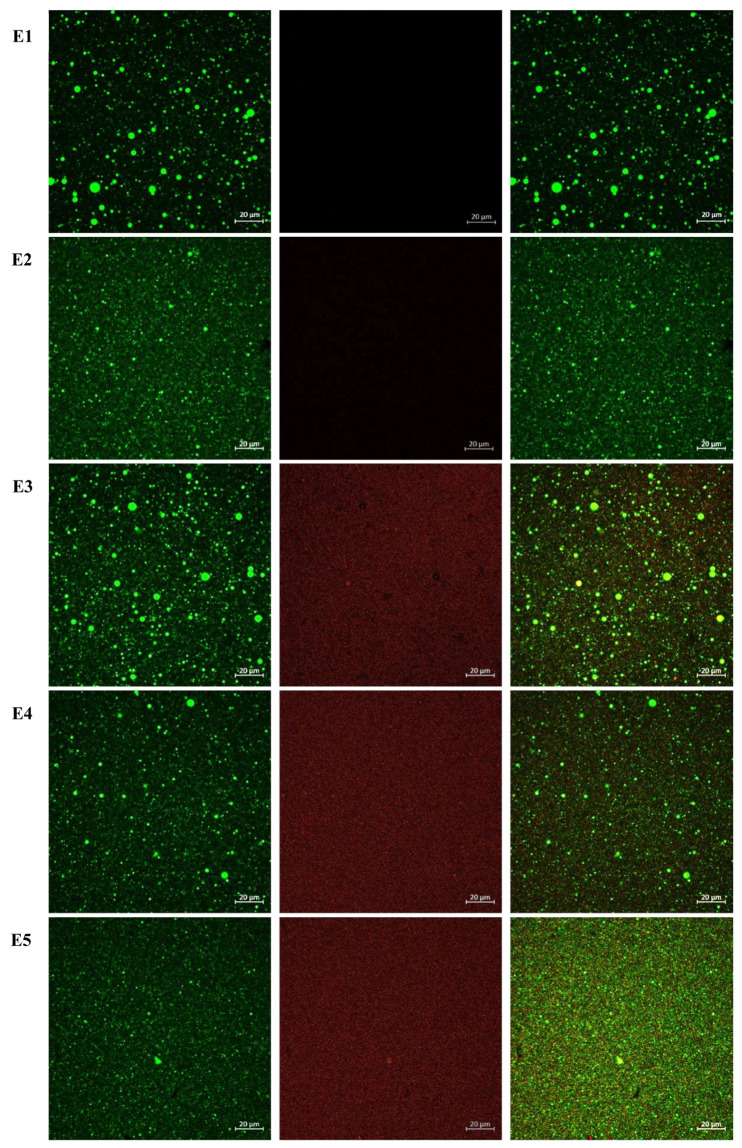
CLSM of DHA oil-in-water nanoemulsions. Oil was stained with Nile Red and Ac was labeled with Nile Blue. Oil droplets were stained with Nile Red (green) and Ac extract with Nile Blue (red). Panels show oil only (**left**), extract only ((**middle**); absent in E1 and E2), and combined staining (**right**). E1 (negative control, no antioxidant), E2 (EDTA, positive control), E3 (Ac at 0.61 mg/mL, 0.5 × IC_50_), E4 (Ac at 1.22 mg/mL, IC_50_), and E5 (Ac at 2.44 mg/mL, 2 × IC_50_).

The microstructure of DHA nanoemulsions was examined by CLSM (Figure 4), revealing clear differences in interfacial coverage and droplet organization among the samples as a function of Ac presence and concentration. All nanoemulsions exhibit uniform droplet distribution and no evidence of flocculation or coalescence, supporting the droplet size and stability data presented in Table 2. Nanoemulsions containing Ac extract (E3-E5) displayed increasing red fluorescence intensity at the droplet interface with rising extract concentration. At the lowest Ac level (E3), partial coverage was observed, with red fluorescence appearing discontinuous around some droplets, suggesting limited adsorption of extract components at the oil–water interface. At intermediate concentration (E4), interfacial labeling was more pronounced and continuous, indicating improved coverage. At the highest concentration (E5), dense and uniform red fluorescence surrounded the droplets, confirming substantial adsorption of extract-derived proteins or other amphiphilic molecules. This progressive increase in interfacial labeling aligns with the dose-dependent incorporation of Ac extract. Overall, CLSM analysis confirms that Ac extract adsorbs at the interface in a concentration-dependent manner. This interfacial association may play a role in antioxidant performance without compromising physical stability, supporting the suitability of Ac for further oxidative stability evaluation.

### 3.4. Oxidative Stability of Nanoemulsion

#### 3.4.1. PV

Oxidative stability was evaluated by monitoring PV over 8 days under FeSO_4_-induced accelerated oxidation, providing a stringent test of antioxidant efficacy in DHA-rich nanoemulsions (Table 4). At Day 0, the negative control (E1) exhibited a significantly higher PV than all other formulations (*p* < 0.05) (PV of the oil was 0.18 meq/kg), indicating extensive hydroperoxide formation during emulsification when no antioxidant protection was present. This pronounced susceptibility can be attributed to the combined effects of high interfacial area and iron-catalyzed radical generation, which rapidly consumed endogenous tocopherols and initiated uncontrolled lipid oxidation. Similar behavior has been reported in omega-3-rich nanoemulsions subjected to prooxidant conditions, where iron ions markedly accelerate primary oxidation processes [45]. Throughout storage, E1 showed a sharp and continuous increase in PV, reaching values exceeding 350 meq/kg oil by Day 8, which were significantly higher than those of all other treatments at each corresponding time point (*p* < 0.05). It is important to note that some PVs approach or exceed the reliable range of the ferric thiocyanate assay and should be interpreted with caution, although overall trends remain reliable. This severe oxidation was accompanied by rapid depletion of α- and γ-tocopherols (Table 4), consistent with the sequential consumption of tocopherols under intense oxidative stress [34]. In contrast, the EDTA-containing nanoemulsion (E2) exhibited significantly lower PVs than E1 and Ac-treated samples across the entire storage period (*p* < 0.05). EDTA strongly inhibited FeSO_4_-driven oxidation by chelating iron, reducing hydroperoxide formation and tocopherol loss, highlighting metal chelation as an effective stabilization strategy for DHA-rich nanoemulsions. Nanoemulsions containing Ac extract (E3–E5) showed significantly improved oxidative stability compared to the negative control (*p* < 0.05 at all time points beyond Day 0), demonstrating that the extract conferred measurable protection even under iron-induced oxidative stress. However, PVs in these samples remained significantly higher than in E2 (*p* < 0.05), indicating that Ac extract could not fully counteract FeSO_4_-driven oxidation. This suggests that the antioxidant mechanisms of Ac extract were less effective against metal-catalyzed initiation than those of EDTA. Among the extract-treated formulations, a concentration-dependent trend emerged during later storage stages. Although PV increased in all Ac-containing nanoemulsions, E4 and particularly E5 tended to exhibit lower PVs than E3 at Day 8, although these differences were not significant (*p* > 0.05).

#### 3.4.2. Tocopherol Consumption

Changes in α-, γ-, and δ-tocopherols were monitored in the nanoemulsions under iron-induced oxidation conditions (Table 4). The initial profile mirrored the oil used, being rich in γ- and δ-tocopherols with low α-tocopherol, while β-tocopherol was not detected, consistent with its negligible level in the parent oil [34]. Therefore, subsequent discussion focuses on α-, γ-, and δ-tocopherols. At Day 0, measurable amounts of α- and γ-tocopherols were detected in all antioxidant-containing nanoemulsions, whereas these tocopherols were either absent or present at markedly lower levels in the negative control (E1), indicating rapid consumption during emulsification when no antioxidant protection was provided. Except for δ-tocopherol, tocopherol contents in E1 remained negligible throughout storage, consistent with severe oxidative stress promoted by ferrous ions and the absence of added antioxidants. In contrast, the EDTA-containing nanoemulsion (E2) preserved tocopherols to a significantly greater extent than all other treatments during storage (*p* < 0.05). Only minor decreases in γ- and δ-tocopherol contents were observed over time, while α-tocopherol levels remained stable. This behavior highlights the effectiveness of EDTA in limiting iron-catalyzed initiation reactions, thereby reducing the demand on endogenous tocopherols as chain-breaking antioxidants. Similar protective effects of metal chelators on tocopherol preservation have been reported previously in omega-3-rich emulsified systems [45].

Nanoemulsions containing Ac extract (E3–E5) exhibited intermediate tocopherol stability between E1 and E2. Tocopherol depletion was significantly slower than in the negative control (*p* < 0.05) but more pronounced than in the EDTA-stabilized nanoemulsion. Across storage, α-tocopherol was the first to decline, followed by γ- and then δ-tocopherol, consistent with the established sequential depletion order of tocopherols during lipid oxidation [34]. Interestingly, this pattern differed in the EDTA-containing emulsion, where only δ-tocopherol declined while α- and γ-tocopherol remained largely unchanged. This behavior may be attributed to the strong metal-chelating activity of EDTA, which effectively suppressed iron-catalyzed oxidation and preserved the more reactive α- and γ-tocopherols. δ-Tocopherol, being less reactive, may have continued to participate in slower radical-trapping reactions or undergo degradation through alternative pathways, highlighting the influence of metal chelation on tocopherol oxidation dynamics. The pattern indicates that tocopherols actively scavenged radicals under iron-induced oxidative stress. Higher Ac concentrations generally accelerated α- and γ-tocopherols depletion, particularly at later times, although differences among Ac-treated samples were not always significant (*p* > 0.05). This accelerated consumption may suggest that tocopherols were more actively engaged in redox processes in the presence of higher extract concentrations, potentially due to interactions between extract constituents and endogenous antioxidants. Despite the enhanced tocopherol depletion observed at higher extract concentrations, δ-tocopherol remained relatively stable across all Ac-treated nanoemulsions, reflecting its lower reactivity and its role as a later-stage antioxidant. The persistence of δ-tocopherol may contribute to residual oxidative protection once more reactive tocopherols are exhausted. Overall, these results demonstrate that Ac extract partially protected endogenous tocopherols compared with the negative control but was less effective than EDTA. The findings further indicate that extract concentration influences tocopherol consumption patterns, highlighting the complex and concentration-dependent interactions between natural extracts and endogenous antioxidants in omega-3 emulsified systems.

#### 3.4.3. Volatile Secondary Oxidation Products

**Figure 5:**
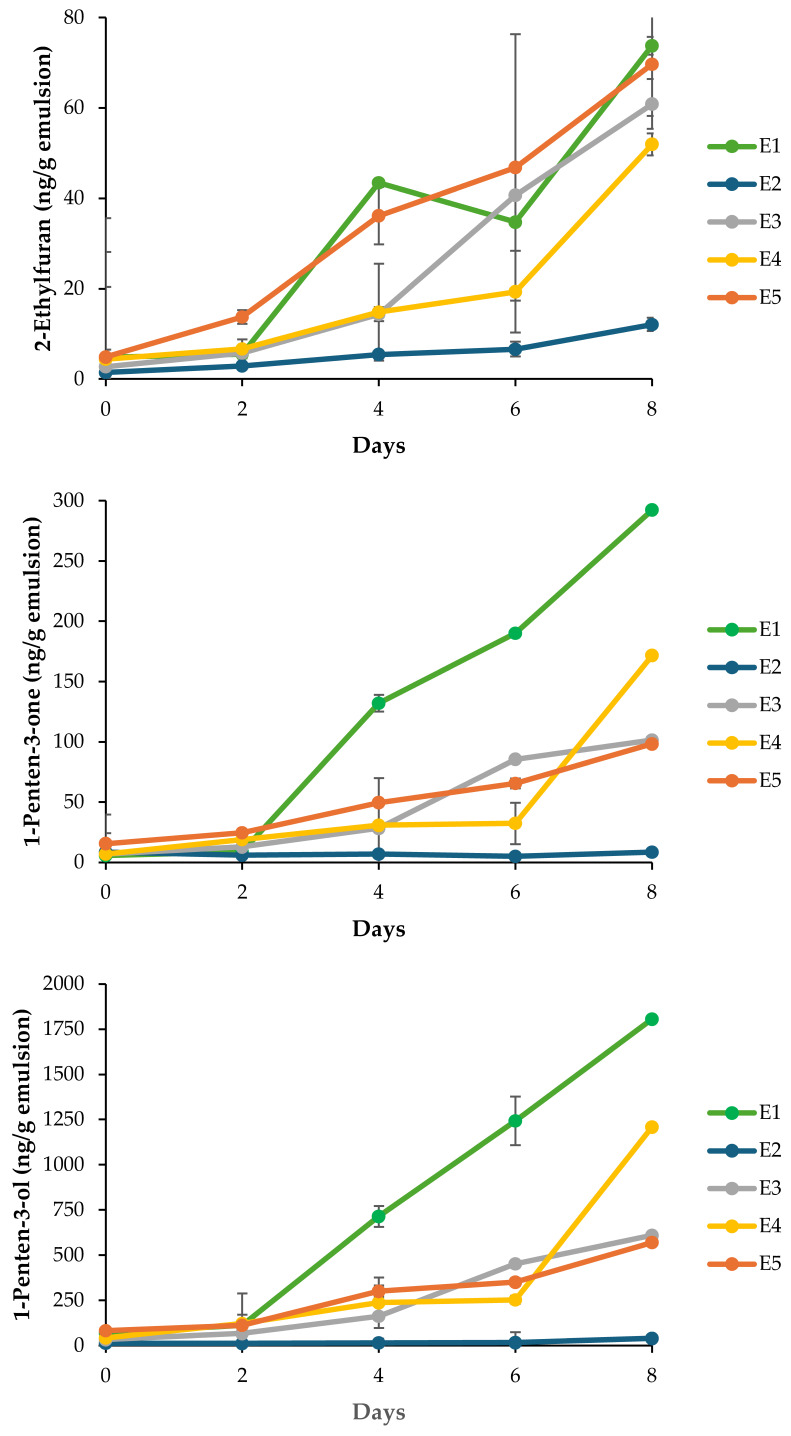

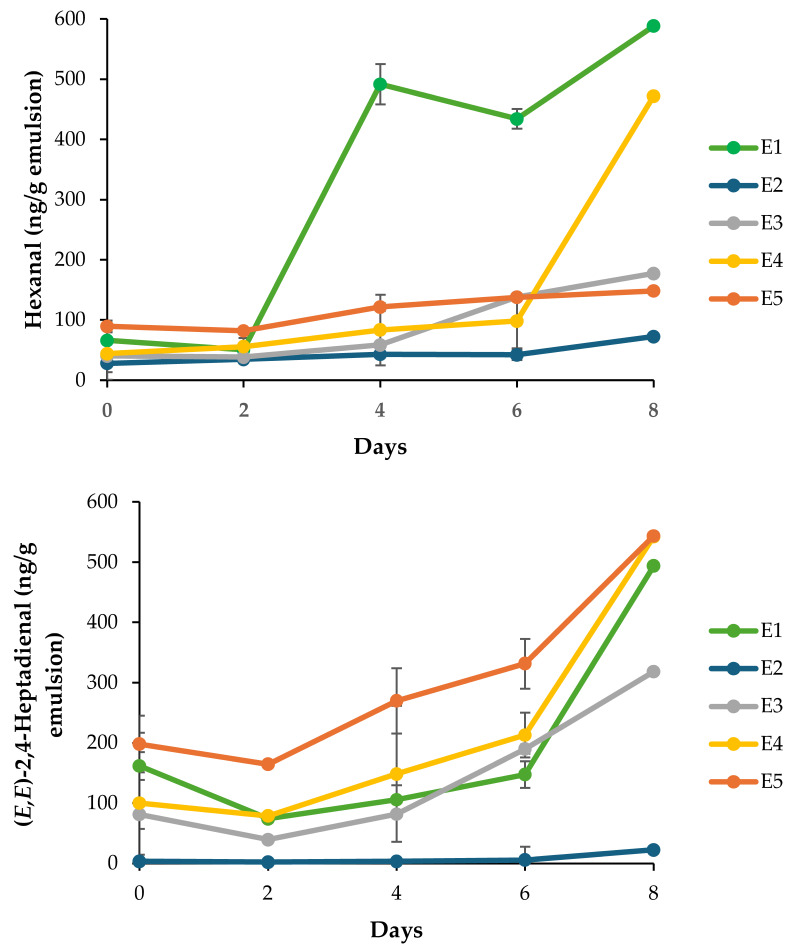
Secondary oxidation volatile compounds of DHA oil-in-water nanoemulsions (n = 3) in an 8-day storage: E1 (negative control, no antioxidant), E2 (EDTA, positive control), E3 (Ac at 0.61 mg/mL, 0.5× IC_50_), E4 (Ac at 1.22 mg/mL, IC_50_), and E5 (Ac at 2.44 mg/mL, 2× IC_50_).

Five key volatiles, i.e., 2-ethylfuran, 1-penten-3-ol, 1-penten-3-one, hexanal, and (*E,E*)-2,4-heptadienal, were monitored (Figure 5) as well-established markers of omega-3 PUFA oxidation [34]. Across all monitored volatiles, the negative control (E1) exhibited the most pronounced increases, confirming extensive secondary oxidation in the absence of antioxidant protection. For instance, 1-penten-3-ol concentrations reached approximately 1750 ng/g by Day 8 in E1, whereas emulsions containing EDTA (E2) maintained levels below 250 ng/g throughout storage. This marked contrast indicates the dominant role of iron-catalyzed radical initiation and highlights the effectiveness of metal chelation in suppressing both primary and secondary oxidation pathways. E3-E5 showed intermediate behavior, with volatile levels significantly lower than those in E1 (*p* < 0.05) but higher than in E2, indicating partial protection under strong prooxidant conditions. A concentration-dependent trend was observed for several volatiles, for instance, E5 exhibited lower levels of 1-penten-3-one, 1-penten-3-ol and hexanal compared to E3 and E4 towards the end of the storage period, suggesting that higher extract concentrations confer improved oxidative stability. However, differences among extract-treated samples were not always statistically significant (*p* > 0.05), reflecting the complex and multifactorial nature of antioxidant performance in emulsified systems. This was particularly the case for 2-ethylfuran which exhibited large standard deviations. The reduction in volatile formation with increasing extract concentration, except for (*E,E*)-2,4-heptadienal, correlates with CLSM observations of interfacial structure (Figure 4). CLSM images revealed progressively enhanced adsorption of Ac extract components at the oil–water interface, ranging from partial coverage in E3 to dense and uniform interfacial labeling in E5. Such interfacial accumulation likely forms a physical barrier that limits prooxidant access while localizing antioxidant molecules at the primary sites of radical initiation [46]. This interfacial stabilization provides a plausible explanation for the improved suppression of volatile formation observed in E5 relative to E3 and E4, despite identical oxidative stress conditions. Nevertheless, the persistence of measurable volatile formation in Ac-treated emulsions compared to EDTA-stabilized systems indicates that interfacial antioxidant mechanisms alone are insufficient to fully counteract metal-driven oxidation. This limitation is further supported by tocopherol depletion patterns, in which higher extract concentrations were associated with accelerated α- and γ-tocopherol consumption (Table 4), possibly reflecting synergistic or competing redox interactions. Collectively, these findings suggest that while interfacial adsorption enhances antioxidant efficacy, further optimization of extract concentration and formulation strategies may be required to achieve protection levels comparable to conventional antioxidants.

## 4. Conclusions

This study demonstrated that extracts from Palmaria palmata, obtained through sequential enzymatic–alkaline extraction, exhibit strong Fe^2+^-chelating activity and can improve oxidative stability of DHA-rich nanoemulsions without compromising physical properties. The selected extract reduced hydroperoxide formation, delayed tocopherol depletion, and lowered volatile oxidation markers in a concentration-dependent manner, while confocal microscopy confirmed interfacial adsorption of bioactive components. Although these natural extracts offer promising clean-label functionality, EDTA remained more effective under severe prooxidant conditions. Future work should focus on optimizing extract composition and delivery strategies to enhance antioxidant performance and fully replace synthetic chelators in functional food applications.

## Figures and Tables

**Table 1:** Protein content (% dry matter), protein recovery (%), and degree of hydrolysis (DH, %) of macroalgal extracts obtained through sequential enzymatic and alkaline procedure.

Treatment	Protein in Extracts (%)	Protein in Solid Residues (%)	Protein Recovery in Extracts (%)	Protein Recovery in Solid Residues (%)	DH (%)
** NoE **	6.41 ± 0.16 ^d^	20.59 ± 1.24 ^ab^	53.65 ± 0.87 ^e^	45.97 ± 1.37 ^a^	24.66 ± 4.84 ^c^
**Ac**	10.11 ± 0.15 ^a^	14.17 ± 0.79 ^c^	94.99 ± 1.40 ^a^	4.97 ± 1.15 ^e^	30.90 ± 3.22 ^bc^
**Fz**	6.70 ± 0.29 ^d^	21.45 ± 0.91 ^a^	61.13 ± 1.78 ^d^	36.61 ± 1.48 ^b^	28.81 ± 4.45 ^bc^
**Fp**	7.89 ± 0.29 ^c^	19.32 ± 1.21 ^ab^	69.88 ± 3.00 ^c^	27.20 ± 2.16 ^c^	22.91 ± 4.31 ^c^
**Ac-Fz**	9.05 ± 0.06 ^b^	17.70 ± 0.44 ^b^	82.13 ± 2.13 ^b^	15.64 ± 2.18 ^d^	45.76 ± 8.36 ^a^
**Ac-Fp**	9.14 ± 0.14 ^b^	17.79 ± 1.72 ^b^	81.41 ± 0.93 ^b^	17.20 ± 2.70 ^d^	29.06 ± 4.44 ^bc^
**Fz-Fp**	7.71 ± 0.04 ^c^	20.82 ± 1.11 ^ab^	68.81 ± 0.36 ^c^	30.92 ± 0.38 ^c^	34.50 ± 4.07 ^b^

Data are presented as mean ± standard deviation. Protein content and recovery were determined in triplicate (n = 3), while DH was determined in eight replicates (n = 8). Different superscript letters indicate significant differences among treatments (*p* < 0.05). NoE (no enzyme), Ac (Alcalase^®^), Fz (Flavourzyme^®^), Fp (Formea^®^ Prime), Ac-Fz (Alcalase^®^ + Flavourzyme^®^), Ac-Fp (Alcalase^®^ + Formea^®^ Prime), and Fz-Fp (Flavourzyme^®^ + Formea^®^ Prime), each followed by alkaline extraction.

**Table 2:** Amino acid composition (mg/g dry matter) of macroalgal extracts obtained through sequential enzymatic and alkaline procedure.

	NoE	Ac	Fz	Fp	Ac-Fz	Ac-Fp	Fz-Fp
**Phenylalanine**	0.74 ± 0.40 ^a^	2.40 ± 1.57 ^a^	1.07 ± 0.58 ^a^	0.74 ± 0.26 ^a^	1.38 ± 0.25 ^a^	1.55 ± 0.29 ^a^	0.86 ± 0.61 ^a^
**Leucine**	1.63 ± 0.02 ^d^	6.80 ± 0.15 ^a^	1.26 ± 0.02 ^d^	0.66 ± 0.31 ^e^	3.34 ± 0.04 ^c^	4.96 ± 0.11 ^b^	1.37 ± 0.16 ^d^
**Isoleucine**	0.97 ± 0.08 ^d^	3.96 ± 0.16 ^a^	0.81 ± 0.07 ^d^	0.70 ± 0.05 ^d^	1.89 ± 0.17 ^c^	3.12 ± 0.45 ^b^	0.99 ± 0.41 ^d^
**Methionine**	0.57 ± 0.09 ^bc^	2.06 ± 0.05 ^a^	0.46 ± 0.04 ^c^	0.46 ± 0.06 ^c^	0.93 ± 0.10 ^b^	1.62 ± 0.04 ^a^	0.68 ± 0.35 ^bc^
**Tyrosine**	0.80 ± 0.15 ^cd^	3.06 ± 0.11 ^a^	0.68 ± 0.17 ^cd^	0.52 ± 0.19 ^d^	1.27 ± 0.20 ^bc^	1.82 ± 0.18 ^b^	0.85 ± 0.39 ^cd^
**Proline**	3.05 ± 0.04 ^c^	5.48 ± 0.08 ^a^	0.79 ± 0.02 ^d^	0.61 ± 0.01 ^d^	3.86 ± 0.05 ^b^	4.42 ± 0.14 ^b^	1.09 ± 0.48 ^d^
**Valine**	1.36 ± 0.09 ^d^	5.78 ± 0.20 ^a^	1.06 ± 0.04 ^d^	0.89 ± 0.04 ^d^	3.00 ± 0.09 ^c^	4.36 ± 0.17 ^b^	1.59 ± 0.73 ^d^
**Alanine**	3.00 ± 0.25 ^c^	8.13 ± 0.22 ^a^	1.58 ± 0.28 ^d^	1.11 ± 0.07 ^d^	5.27 ± 0.03 ^b^	6.06 ± 0.30 ^b^	2.02 ± 0.83 ^cd^
**Threonine**	0.81 ± 0.07 ^cd^	3.75 ± 0.28 ^a^	1.39 ± 1.03 ^bcd^	0.58 ± 0.05 ^d^	2.07 ± 0.28 ^bc^	2.74 ± 0.04 ^ab^	1.09 ± 0.48 ^cd^
**Glycine**	2.85 ± 0.25 ^cd^	5.76 ± 0.50 ^a^	1.97 ± 0.24 ^cd^	1.41 ± 0.24 ^d^	3.48 ± 1.12 ^bc^	4.96 ± 0.75 ^ab^	1.48 ± 0.61 ^d^
**Serine**	2.07 ± 0.73 ^bc^	5.40 ± 0.85 ^a^	1.72 ± 0.37 ^bc^	1.30 ± 0.83 ^c^	3.62 ± 0.42 ^ab^	4.68 ± 0.21 ^a^	2.27 ± 0.91 ^bc^
**Arginine**	2.25 ± 0.19 ^b^	6.51 ± 0.09 ^a^	2.19 ± 0.03 ^b^	1.96 ± 0.05 ^b^	2.97 ± 0.11 ^b^	6.23 ± 0.24 ^a^	3.02 ± 1.19 ^b^
**Histidine**	0.78 ± 0.30 ^a^	1.23 ± 0.15 ^a^	0.76 ± 0.30 ^a^	0.69 ± 0.47 ^a^	0.60 ± 0.25 ^a^	1.54 ± 0.23 ^a^	0.63 ± 0.39 ^a^
**Lysine**	1.34 ± 0.08 ^b^	4.00 ± 0.27 ^a^	1.35 ± 0.21 ^b^	1.14 ± 0.39 ^b^	1.99 ± 0.41 ^b^	4.37 ± 0.51 ^a^	2.06 ± 1.24 ^b^
**Glutamic acid**	7.45 ± 0.04 ^d^	13.94 ± 0.03 ^a^	2.43 ± 0.21 ^e^	2.28 ± 0.21 ^e^	9.80 ± 0.30 ^c^	12.05 ±0.58 ^b^	2.98 ± 1.04 ^e^
**Cystine**	5.98 ± 0.64 ^f^	7.35 ± 0.99 ^ef^	78.24 ±2.38 ^b^	68.92 ±1.88 ^c^	11.31 ± 1.03 ^de^	15.47 ±1.22 ^d^	83.73 ± 1.09 ^a^
**Aspartic acid**	7.55 ± 0.83 ^bc^	14.59 ± 1.13 ^a^	4.58 ± 0.66 ^c^	4.09 ± 0.60 ^c^	9.66 ± 1.38 ^b^	13.73 ±1.33 ^a^	5.05 ± 2.12 ^c^
**TAA**	43.20 ±1.59 ^d^	100.19 ± 2.83 ^ab^	102.33 ± 1.95 ^ab^	88.07 ± 1.89 ^b^	66.42 ±2.68 ^c^	93.68 ±3.93 ^b^	111.77 ± 10.86 ^a^
**EAA**	9.00 ± 0.32 ^d^	33.03 ± 1.61 ^a^	8.84 ± 0.87 ^d^	6.39 ± 0.62 ^d^	16.47 ±0.69 ^c^	26.08 ±1.35 ^b^	10.12 ± 4.23 ^d^
**EAA/TAA**	0.21 ± 0.01 ^c^	0.33 ± 0.01 ^a^	0.09 ± 0.01 ^d^	0.07 ± 0.01 ^d^	0.25 ± 0.01 ^b^	0.28 ± 0.01 ^b^	0.09 ± 0.03 ^d^

Data are presented as mean ± standard deviation (n = 3). Different superscript letters indicate significant differences among treatments (*p* < 0.05). NoE (no enzyme), Ac (Alcalase^®^), Fz (Flavourzyme^®^), Fp (Formea^®^ Prime), Ac-Fz (Alcalase^®^ + Flavourzyme^®^), Ac-Fp (Alcalase^®^ + Formea^®^ Prime), and Fz-Fp (Flavourzyme^®^ + Formea^®^ Prime), each followed by alkaline extraction. TAA and EAA represent total amino acids and essential amino acids, respectively.

**Table 3:** Zeta potential, droplet size, and apparent viscosity of DHA oil-in-water nanoemulsions.

	ζ-Potential (mV) Day 1	D_3,2_ (nm) Day 1	D_3,2_ (nm) Day 8	D_4,3_ (nm) Day 1	D_4,3_ (nm) Day 8	Viscosity (cP) Day 1	Viscosity (cP) Day 8
**E1**	−17.30 ± 0.42 ^a^	79.90 ± 0.14 ^a^	78.15 ± 0.07 ^b^	205.0 ± 0.0 ^b^	204.5 ± 0.71 ^b^	1.34 ± 0.06 ^a^	1.16 ± 0.05 ^a^
**E2**	−18.95 ± 0.21 ^a^	79.90 ± 0.28 ^a^	78.55 ± 0.07 ^b^	215.0 ± 1.41 ^a^	212.0 ± 0.0 ^a^	1.40 ± 0.09 ^a^	1.39 ± 0.09 ^a^
**E3**	−17.30 ± 0.14 ^a^	80.25 ± 0.35 ^a^	80.40 ± 0.28 ^a^	214.5 ± 2.12 ^a^	212.0 ± 1.41 ^a^	1.44 ± 0.10 ^a^	1.36 ± 0.06 ^a^
**E4**	−17.25 ± 0.49 ^a^	78.35 ± 0.21 ^b^	77.05 ± 0.21 ^c^	199.0 ± 1.41 ^b^	197.5 ± 0.71 ^c^	1.47 ± 0.10 ^a^	1.41 ± 0.01 ^a^
**E5**	−18.40 ± 0.57 ^a^	77.65 ± 0.07 ^b^	75.85 ± 0.21 ^d^	201.5 ± 0.71 ^b^	198.0 ± 0.0 ^c^	1.43 ± 0.10 ^a^	1.34 ± 0.05 ^a^

Data are presented as mean ± standard deviation (n = 2). Different superscript letters indicate significant differences among nanoemulsions at each sampling day (*p* < 0.05). E1 (negative control, no antioxidant), E2 (EDTA, positive control), E3 (Ac at 0.61 mg/mL, 0.5 × IC_50_), E4 (Ac at 1.22 mg/mL, IC_50_), and E5 (Ac at 2.44 mg/mL, 2 × IC_50_).

**Table 4:** Peroxide value (PV) and tocopherol content of DHA nanoemulsions during an 8-day storage.

Sample	Day	Peroxide Value (meq/kg oil)	α-Tocopherol (µg/g Emulsion)	γ-Tocopherol (µg/g Emulsion)	δ-Tocopherol (µg/g Emulsion)
**E1**	Day 0	99.43 ± 26.45 ^b,w^	3.27 ± 0.13 ^a,x^	0.00 ± 0.00 ^a,x^	25.34 ± 1.00 ^a,x^
	Day 2	118.85 ± 4.68 ^b,y^	2.98 ± 0.79 ^a,w^	0.00 ± 0.00 ^a,z^	20.71 ± 0.78 ^ab,x^
	Day 4	289.44 ± 8.52 ^a,w^	3.36 ± 0.99 ^a,w^	0.00 ± 0.00 ^a,z^	23.32 ± 1.55 ^a,w^
	Day 6	336.93 ± 20.14 ^a,w^	2.98 ± 0.13 ^a,y^	0.00 ± 0.00 ^a,z^	10.21 ± 1.06 ^c,y^
	Day 8	357.15 ± 24.36 ^a,w^	2.58 ± 0.24 ^a,x^	0.00 ± 0.00 ^a,z^	17.48 ± 1.11 ^b,w^
**E2**	Day 0	21.33 ± 0.43 ^b,xy^	8.76 ± 0.00 ^b,w^	37.06 ± 0.14 ^a,w^	38.54 ± 0.74 ^a,w^
	Day 2	16.62 ± 1.38 ^b,z^	9.07 ± 0.41 ^ab,w^	36.01 ± 0.21 ^a,w^	39.81 ± 2.78 ^a,w^
	Day 4	22.17 ± 1.59 ^b,y^	10.51 ± 0.22 ^a,w^	35.75 ± 1.35 ^a,w^	14.37 ± 0.47 ^b,x^
	Day 6	34.38 ± 2.15 ^a,y^	10.38 ± 0.02 ^a,w^	34.16 ± 0.55 ^a,w^	13.76 ± 0.17 ^b,w^
	Day 8	32.00 ± 1.00 ^a,y^	10.49 ± 0.52 ^a,w^	34.27 ± 1.77 ^a,w^	13.81 ± 0.65 ^b,x^
**E3**	Day 0	41.67 ± 3.60 ^c,wxy^	10.20 ± 2.44 ^a,w^	27.18 ± 7.78 ^a,w^	11.38 ± 3.07 ^a,y^
	Day 2	113.73 ± 2.74 ^b,y^	9.53 ± 1.78 ^a,w^	31.17 ± 0.08 ^a,x^	13.52 ± 0.19 ^a,y^
	Day 4	126.71 ± 1.74 ^ab,x^	8.65 ± 1.91 ^a,w^	30.03 ± 0.21 ^a,x^	13.49 ± 0.04 ^a,wx^
	Day 6	158.10 ± 28.22 ^ab,x^	7.68 ± 1.74 ^ab,wx^	26.12 ± 1.61 ^a,x^	12.87 ± 0.19 ^a,x^
	Day 8	186.63 ± 5.08 ^a,x^	0.00 ± 0.00 ^b,x^	15.08 ± 0.85 ^a,xy^	11.65 ± 0.28 ^a,xy^
**E4**	Day 0	11.39 ± 1.70 ^b,y^	8.27 ± 0.66 ^a,wx^	31.00 ± 1.17 ^a,w^	13.39 ± 0.40 ^a,y^
	Day 2	183.92 ± 2.75 ^a,w^	6.49 ± 0.43 ^ab,w^	27.88 ± 0.40 ^a,xy^	12.81 ± 0.00 ^ab,y^
	Day 4	156.38 ± 7.69 ^a,x^	4.51 ± 1.36 ^ab,w^	21.66 ± 0.90 ^ab,xy^	12.31 ± 0.37 ^ab,x^
	Day 6	127.24 ± 37.95 ^a,xy^	4.95 ± 0.46 ^ab,xy^	24.10 ± 1.42 ^bc,x^	12.59 ± 0.01 ^ab,wx^
	Day 8	133.95 ± 8.12 ^a,x^	3.34 ± 1.50 ^b,x^	19.62 ± 1.36 ^c,x^	12.12 ± 0.19 ^b,xy^
**E5**	Day 0	77.93 ± 10.56 ^a,wx^	8.73 ± 0.07 ^a,w^	30.39 ± 0.02 ^a,w^	13.26 ± 0.11 ^a,y^
	Day 2	152.38 ± 9.94 ^a,x^	3.75 ± 3.71 ^a,w^	23.51 ± 0.83 ^b,y^	11.70 ± 0.40 ^b,y^
	Day 4	134.79 ± 35.92 ^a,x^	3.27 ± 2.97 ^a,w^	19.47 ± 0.76 ^c,y^	11.23 ± 0.13 ^b,x^
	Day 6	153.61 ± 1.31 ^a,x^	5.33 ± 0.00 ^a,xy^	19.64 ± 0.31 ^c,y^	11.20 ± 0.15 ^b,xy^
	Day 8	129.52 ± 20.58 ^a,x^	0.00 ± 0.00 ^a,x^	13.49 ± 0.52 ^d,y^	10.29 ± 0.54 ^b,y^

Data are presented as mean ± standard deviation (n = 2). The superscripts a–d indicate significant differences among the samples at each time point, and the superscripts w–z indicate significant differences among the time points for each sample (*p* < 0.05). E1 (negative control, no antioxidant), E2 (EDTA, positive control), E3 (Ac at 0.61 mg/mL, 0.5 × IC_50_), E4 (Ac at 1.22 mg/mL, IC_50_), and E5 (Ac at 2.44 mg/mL, 2 × IC_50_).

## Data Availability

The original contributions presented in this study are included in the article. Further inquiries can be directed to the corresponding authors.
